# Alterations of gray matter volume and functional connectivity in patients with cognitive impairment induced by occupational aluminum exposure: a case-control study

**DOI:** 10.3389/fneur.2024.1500924

**Published:** 2025-01-07

**Authors:** Huaxing Meng, Bo Liu, Xiaoting Lu, Yan Tan, Shanshan Wang, Baolong Pan, Hui Zhang, Qiao Niu

**Affiliations:** ^1^Department of Neurology, First Hospital of Shanxi Medical University, Taiyuan, China; ^2^College of Medical Imaging, Shanxi Medical University, Taiyuan, China; ^3^School of Public Health, Shanxi Medical University, Taiyuan, China; ^4^Science and Education Department, Sixth Hospital of Shanxi Medical University, Taiyuan, China

**Keywords:** mild cognitive impairment, plasma aluminum, gray matter volume, functional connectivity, aluminum

## Abstract

**Background:**

Cognitive impairment (CI) is a condition in which an individual experiences noticeable impairment in thinking abilities. Long-term exposure to aluminum (Al) can cause CI. This study aimed to determine the relationship between CI and MRI-related changes in postroom workers exposed to Al.

**Methods:**

Thirty patients with CI and 25 healthy controls were recruited. Plasma aluminum levels were measured using inductively coupled plasma-mass spectrometry. Cognitive function was assessed using the Montreal Cognitive Assessment (MoCA) and an auditory-verbal learning test (AVLT). All participants underwent magnetic resonance imaging scans. 3D T1-weighted anatomical images and resting-state functional magnetic resonance imaging data were acquired, and voxel-based morphometry and ROI-based FC were used for analysis. A mediation analysis was also conducted.

**Results:**

Plasma aluminum levels were significantly higher in the CI group than in the normal control group. The gray matter (GM) volume in the left caudate and bilateral hippocampus was lower in the CI group and was positively correlated with cognitive scale scores. There was no significant difference in functional connectivity (FC) between the left caudate and the whole brain between the two groups. Significant alterations in hippocampal FC were observed in certain brain areas, mainly in the left cerebellar vermis, left middle frontal gyrus (BA9), and right superior frontal gyrus relative to the supplementary motor area (BA6). The FC coefficients were also associated with cognitive scale scores. Furthermore, plasma Al concentration was negatively correlated with the Montreal Cognitive Assessment score, bilateral hippocampal GM volume, and FC coefficient between the left hippocampus and left cerebellar vermis. Mediation analysis showed GM alteration of left caudate and bilateral hippocampus and FC alteration of left hippocampus to left cerebellar vermis could explained 19.80–32.07% of the effect of MoCA scores change related to Al exposure, besides the GM alteration of right hippocampus acted as indirect mediator (68.75%) of the association between Al and AVLT delayed recall scores.

**Conclusion:**

Our data indicates that alterations in the structure and function of special brain domain, especially the hippocampus, are associated with Al-induced CI. These brain regions can partly explain the effect of Al on cognitive impairment.

## 1 Introduction

Aluminum (AI) is the third most abundant element in the Earth’s crust and is commonly detected in stable molecular compounds. However, owing to acid rain, industrial activities, and the addition of Al-containing agents to foods, cosmetics, medicines, and vaccines, Al is present in almost all aspects of life. The human body may be exposed to Al through the skin, digestive tract, muscles, or respiratory tract (1). Long-term Al exposure can lead to deposition and damage in the human body (2).

Inhaled ultrafine Al particles can cross the blood-brain barrier and enter the central nervous system via the olfactory nerve, leading to cognitive dysfunction (3). Al-induced cognitive dysfunction is a serious public health concern. The neurotoxicity of Al has been confirmed in several studies. Longstreth et al. (4) first reported a decline in cognitive function among individuals exposed to AI in the workplace. Riihimäki and Aitio (5) performed a meta-analysis in Germany and found a dose-response relation between cognitive function and blood AI (6). Our studies (7–9) have demonstrated that the cognitive function of electrolytic Al workers decreased across multiple cognitive domains measured using various scales. The Montreal Cognitive Assessment Beijing Version (MoCA) (10) is one of the most commonly used screening tool for cognitive functioning. It primarily investigates comprehensive cognitive functions, including visuospatial abilities, executive function, naming, memory, attention, language, abstraction, delayed recall, and orientation. The Auditory Verbal Learning Test (AVLT) (11) is a word-list task commonly used to evaluate episodic memory. Most studies examining workplace exposure to Al and cognitive dysfunction have reported memory impairment (12–14). Our previous longitudinal study (15) showed that continuous exposure to Al in the workplace can impair workers’ overall cognitive abilities, particularly episodic memory function.

With the development of neuroimaging technology, magnetic resonance imaging (MRI) has been used to study the brain structure and functional activities in the human body. 3D T1-weighted anatomical structural MRI can scan thinner tissues and obtain images of more tissue layers, revealing more subtle changes in brain structure and facilitating the identification of early lesions. Resting-state functional MRI (rs-fMRI) is a functional MRI technique that indirectly reflects the activity of local neurons based on blood oxygen level-dependent signals. Studying the pollutome-connectome axis and uncovering the link between the pollution spectrum and cognitive impairment (CI) will pave the way for the development of effective preventive treatments for thousands of people living in polluted situations (16).

CI can be induced by many factors, as demonstrated by neurobehavioral tests. However, the same functional and/or structural variations may underlie seemingly similar CIs caused by different factors. Given that neurobehavioral tests can be affected by subjective and environmental factors, it is better to obtain more objective results via MRI to observe the real functional and structural changes induced by different factors. Al exposure can induce cognitive decline, as evidenced by human investigations and decreased learning and memory in animal experiments. However, changes in brain function and structure induced by Al have not been fully clarified. In recent years, there has been a lack of human research on brain structure and function in individuals with chronic Al-induced cognitive decline. Only one animal study has examined Al-induced structural changes in the brain (17). MRI of maltol aluminum (Al(mal)_3_)-treated New Zealand white rabbits revealed temporal lobe atrophy and ventricular dilatation. The magnetic resonance field used was 0.2 T, the image resolution was low, and there were no functional MRI data. The age at which all workers exhibited CI may have been earlier than that of the general population. It is important to detect abnormal brain structure and function in workers via MRI to guide early intervention.

In the present study, 3D T1-weighted anatomical structural MRI and rs-MRI were used. The purpose of this study was to explore (1) alterations in gray matter (GM) volume and functional connectivity (FC) in patients with CI induced by occupational exposure to aluminum. (2) The relations between plasma Al concentrations, cognitive indices, and functional and structural changes in the brain, even the potential mediating role of these brain regions.

## 2 Materials and methods

### 2.1 Participants

This study was conducted at a large aluminum factory in North China. There were no lead smelters, battery factories, manganese smelters, mercury factories, or pesticide factories near the Al factories. According to our measurements, Al levels in the drinking water of the surrounding area where the workers resided were below the threshold limits (200 μg/L) recommended by the World Health Organization.

All participants were native Chinese speakers. Written informed consents were obtained. Our study was performed in accordance with the Helsinki Declaration, as revised in 2008. This study was performed in accordance with the ethical standards of the Ethics and Human Subjects Committees of Shanxi Medical University (approval code: 2014059). The participants had similar dietary habits (primarily steamed bread and noodles made from wheat flour). Basic demographic and medical information was collected. The participants then underwent face-to-face neuropsychological tests conducted by specialized and experienced neurologists or psychologists.

### 2.2 Cognitive assessment

#### 2.2.1 The MoCA (the Beijing version)

MoCA is one of the most commonly used screening tool for cognitive functioning (10). It can evaluate general cognitive function, including attention and concentration, executive function, memory, language, visual, abstract thinking, calculation and orientation. The total score is 30 points, with ≥26 indicating normal. The lower the score, the more severe the cognitive impairment.

#### 2.2.2 AVLT (Shanghai mental health version)

AVLT is a word-list task commonly used to evaluate episodic memory (11). It can evaluate memory function. There were a total of 15 words, which were read to the participants. They were immediately asked to recall and record the number of correct memories. After 5 min, they would recall for the second time and record the number of correct memories. Conduct the third recall after 20 min to evaluate long term memory. Each correct answer get one point.

The inclusion criteria for the CI (18) group were as follows: (1) right-handed and native Chinese speakers; (2) aged 40–60 years; (3) non-smokers and non-drinkers; (4) no conditions that may cause CI, such as hepatic or renal disorders, cerebrovascular diseases, brain trauma, epilepsy, Parkinson’s disease, or mental disorders; (5) no family history of dementia among first-degree relatives; (6) no history of continuous medication with drugs containing Al, such as antacids affecting the central nervous system; (7) no abnormal signals found by MRI, including T2-weighted dark-fluid and T1-weighted MR images; (8) no vision or hearing impairment; and (9) classification into a CI (MoCA <26) group and a normal control (NC) group (MoCA ≥26) according to their MoCA scores. Additionally, owing to budgetary limitations, 55 participants were ultimately recruited, including 30 patients with CI and 25 NC patients matched for sex, age, and education.

### 2.3 Biological sample collection and determination of plasma Al concentration

Venous blood was collected from the participants in the morning. The plasma Al concentration was measured using inductively coupled plasma-mass spectrometry in accordance with previous laboratory methods (7).

### 2.4 Image acquisition

MRI data were acquired using a 3 Tesla Siemens Magnetom Skyra scanner (Siemens, Erlangen, Germany) at the First Hospital of Shanxi Medical University with a 32-channel RF head coil. Foam padding and tape were applied to minimize head motion. A high-resolution structural image was acquired using a 3D T1-weighted magnetization-prepared rapid gradient echo scan with the following parameters: repetition time = 2,530 ms; echo time = 2.01 ms, flip angle = 18°; matrix = 256 × 256; field of view = 256 × 256 mm^2^; sagittal slices = 192, thickness = 1 mm; and total scan time = 6 min 3 s.

The rs-fMRI images were obtained using an echo-planar imaging sequence with 33 slices parallel to the AC-PC line. The scanning parameters were as follows: repetition time = 2,000 ms, echo time = 30 ms, flip angle = 90°, matrix = 64 × 64, field of view = 224 × 224 mm^2^, thickness = 3.5 mm, and gap = 0.7 mm. The scanning lasted for 8 min (240 volumes), during which all participants were asked to close their eyes, lie down, relax, stay awake, and try not to think about anything during data acquisition.

### 2.5 Image analysis

#### 2.5.1 Voxel-based morphometry analysis

Voxel-based morphometry (VBM) analysis of the brain GM volume of each participant was performed using the Computational Anatomy Toolbox (CAT12.6; http://www.neuro.uni-jena.de/cat) version r1450 implemented in the Statistical Parametric Mapping software (SPM12; http://www.fil.ion.ucl.ac.uk/spm) with the default settings described in the CAT12 manual. T1 images were affine-registered to a European brain template, spatially normalized to the Montreal Neurological Institute template, and segmented into GM, white matter (WM), and cerebrospinal fluid (CSF). The voxel size of the normalized images was 1.5 mm^3^. The GM was then modulated using the Jacobian determinant of spatial transformation, reflecting relative differences in regional GM volume. To ensure image quality, a homogeneity check was performed, and poor-quality data were excluded. The total intracranial volume (TIV) was estimated using CAT12 to correct for differences in brain size in subsequent analyses (19). Finally, the images were smoothed using an 8-mm full-width-at-half-maximum (FWHM) Gaussian kernel. This preprocessing step aimed to prepare the data for subsequent general linear model (GLM) analysis and to mitigate potential misalignments introduced during registration.

For the VBM analysis, two-sample *t*-tests were conducted to identify significant differences in GM volume between the two groups, with age, education, and TIV as covariates in SPM12. Significance levels were set at *p* < 0.001, with cluster-level family-wise error-corrected *p* < 0.05. The mean GM volume in the region detected via VBM analysis was calculated to determine the correlation between the GM volume in the region and participants’ cognitive scores.

#### 2.5.2 rs-fMRI analysis

The rs-fMRI data were preprocessed using the Data Processing & Analysis for Brain Imaging toolbox (V3.1_180801, http://rfmri.org/DPABI) (20) based on SPM12. For each participant, the preprocessing included the following steps: (1) discarding the first 10 volumes of the scanning session to exclude the effect of scanner nonequilibrium; (2) slice timing; (3) motion correction with rigid body translation and rotation parameters; (4) tissue segmentation of 3D-T1 images into WM, GM, and CSF by DARTEL (21); (5) co-registration of 3D-T1 images to rs-fMRI volumes; (6) normalization into Montreal Neurological Institute space; (7) spatial smoothing with a 6-mm full-width-at-half-maximum Gaussian kernel; (8) removing the signal trend with time linearization; (9) bandpass filtering (0.01–0.08 Hz); and (10) regressing out nuisance variables, including the Friston 24-parameter of head motion (22), WM signal, and CSF, to further reduce nonneuronal signal confounds. Three NCs and four patients with CI were excluded from subsequent analysis because of excessive head motion (>3 mm/3°). Finally, rs-fMRI data from 47 participants were included in the analysis: 25 patients with CI and 22 NCs.

For the ROI-based FC analyses, the regions identified in the VBM analysis were selected as seeds to explore altered FC of the ROI and the whole brain between the CI and NC groups using two-sample t-tests with age, education, and mean framewise displacement as covariates in SPM12. As our approach was data-driven and exploratory, to increase sensitivity, we used a relatively liberal threshold (voxel-level *p* < 0.01, cluster-level family-wise error-corrected *p* < 0.05). The mean FC coefficient of the region identified in the FC analysis and the correlation between the FC coefficient of the region and participants’ cognitive scores were extracted.

#### 2.5.3 Statistical analysis

Demographic, clinical, neuropsychological, and behavioral data were analyzed using SPSS 19.0 (SPSS, Chicago, IL, United States). Two-sample *t*-tests and Mann–Whitney *U* tests were used to compare demographic and clinical data between the two groups. The correlation between plasma Al concentrations, brain metrics, and cognitive scores was estimated using partial correlation analysis after adjusting for age, education, and mean framewise displacement (for the FC coefficient)/TIV (for GM volume). The mediation R (version 4.5.0) was used for mediation effect analysis. The threshold value for establishing significance was set at *p* < 0.05.

## 3 Results

### 3.1 Demographic and clinical characteristics

Demographic information is listed in Table 1. All participants were men. There were no significant differences in age or education between the CI and NC groups (*p* > 0.05). The plasma Al concentration in the CI group was higher than that in the NC group. Differences were found in the MoCA and AVLT scores between the two groups, adjusted for age and education, including immediate recall, delayed recall, and recognition scores (Table 1).

**Table 1:** Characteristics of the participants.

	NC group (*n* = 25)	CI group (*n* = 30)	*p*-value
Age (years)	47.20 ± 4.85	47.10 ± 5.54	0.94
Education	9.00 ± 0.87	9.40 ± 1.89	0.31
Plasma Al	15.08 (4.63, 34.77)	38.77 (30.16, 50.34)	<0.001
MoCA scores	27.96 ± 0.93	18.83 ± 2.44	<0.001
AVLT, immediate recall scores	22.64 ± 4.98	17.00 ± 4.80	<0.001
AVLT, delayed recall scores	8.88 ± 2.70	5.77 ± 2.60	<0.001
AVLT, recognition scores	11.80 ± 1.83	9.53 ± 3.16	0.002

The data are presented as the mean ± standard deviation or median (25th, 75th). The unit for plasma Al was μg/L. *n*, number of participants; NC, normal control; CI, cognitive impairment; MoCA, Montreal Cognitive Assessment; AVLT, Auditory Verbal Learning Test.

### 3.2 Correlations between plasma Al concentrations and cognitive scale scores

**Figure 1:**
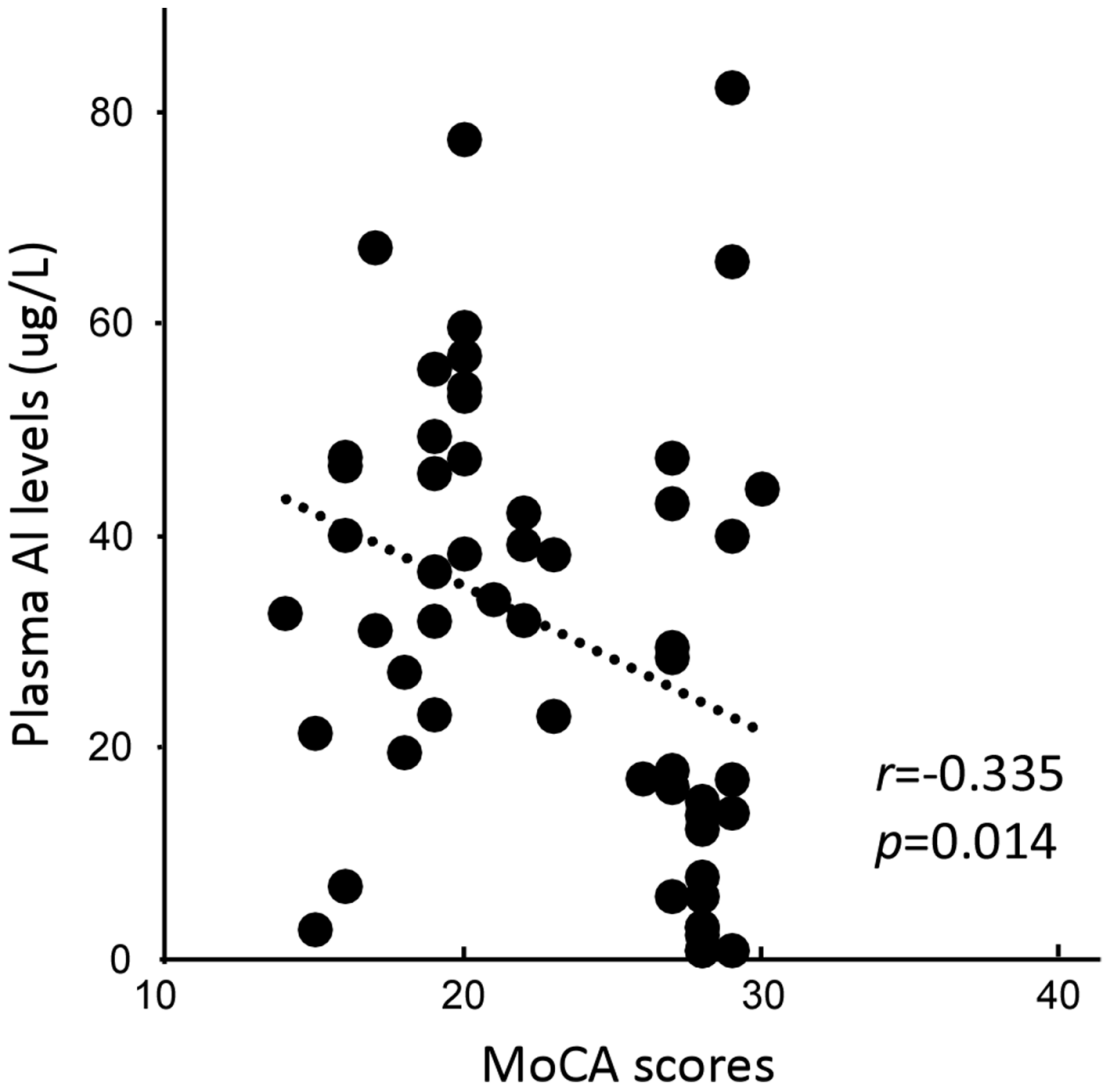
Association between the plasma Al concentration and the Montreal Cognitive Assessment, Beijing version (MoCA) score.

Plasma Al concentrations were negatively correlated with MoCA scores (*r* = −0.335, *p* = 0.014; Figure 1). No significant correlation was found between plasma Al concentration and AVLT scores.

### 3.3 GM volume and correlations with cognitive scales and plasma Al concentrations

**Figure 2:**
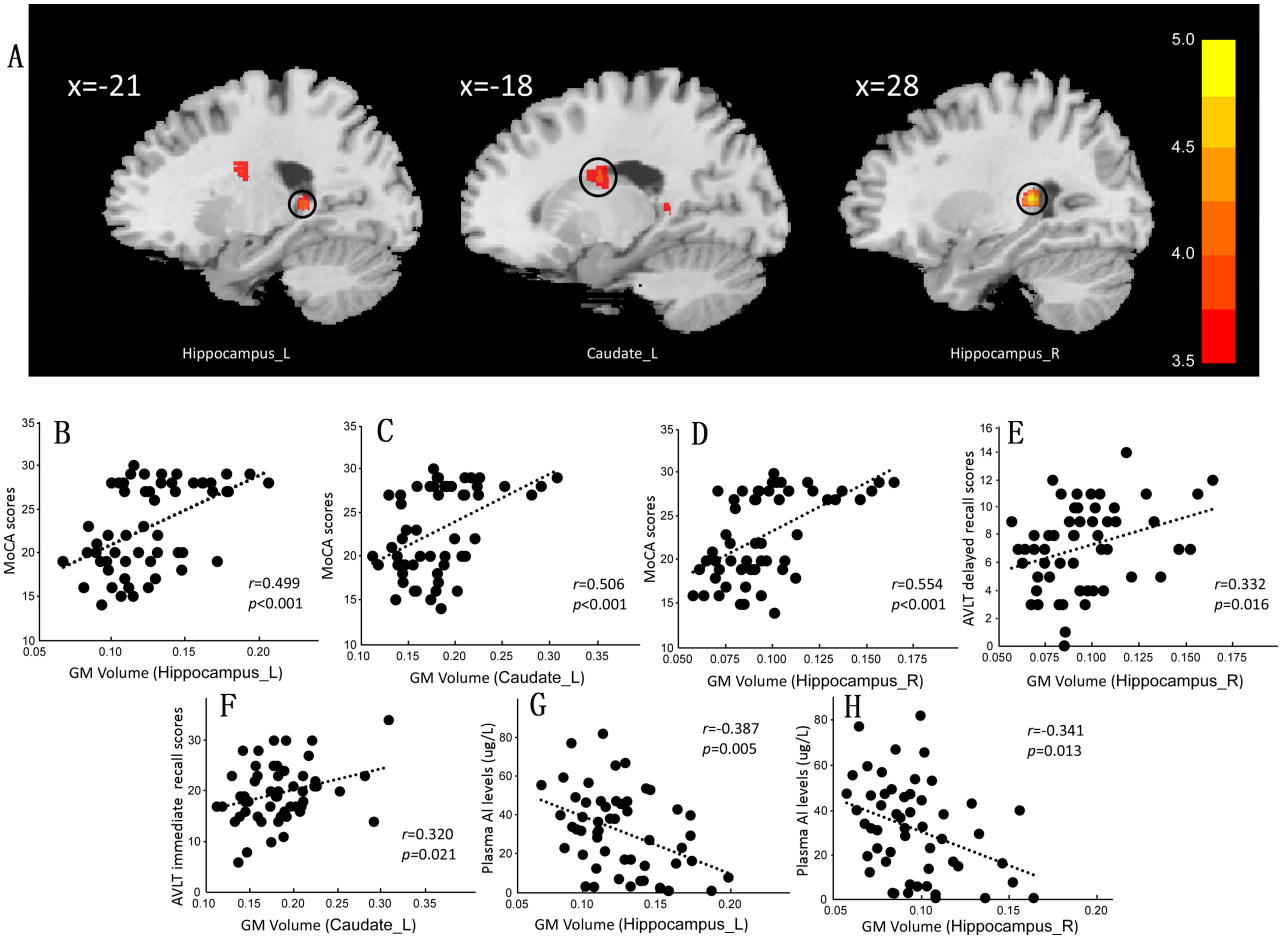
Differences in gray matter (GM) maps between the normal control (NC) group and the cognitive impairment (CI) group. **(A)** Compared with those in the NC group, the GM volume in the CI group was significantly lower in the bilateral hippocampus and left caudate. **(B–F)** The mean GM volume was calculated within the significantly altered brain area of each participant. The GM volume in the bilateral hippocampus and left caudate was positively correlated with the cognitive scale score. **(G,H)** The GM volume in the bilateral hippocampus was also negatively correlated with the plasma Al concentration. MoCA, Montreal Cognitive Assessment, Beijing version; AVLT, Auditory Verbal Learning Test.

Compared with that in NC group, the GM volume in the left caudate and bilateral hippocampus in the CI group was lower (Supplementary Table S1 and Figure 2A). Subsequently, we extracted the mean GM volume for brain areas that were significantly altered in each participant and examined the relation between GM volume and cognitive function. The results showed that the GM volumes of the left hippocampus, left caudate, and right hippocampus were positively correlated with MoCA scores in both groups (*r* = 0.499, *p* < 0.001; Figure 2B; *r* = 0.506, *p* < 0.001; Figure 2C; and *r* = 0.554, *p* < 0.001; Figure 2D). The GM volume in the right hippocampus and left caudate were also positively correlated with AVLT delayed recall and AVLT immediate recall scores (*r* = 0.332, *p* = 0.016; Figure 2E; and *r* = 0.320, *p* = 0.021; Figure 2F, respectively). Plasma Al concentrations were negatively associated with GM volume in both hippocampus (L: *r* = −0.387, *p* = 0.005; Figure 2G; R: *r* = −0.341, *p* = 0.013; Figure 2H).

### 3.4 FC, correlations with cognitive scale scores, and plasma Al concentrations

**Figure 3:**
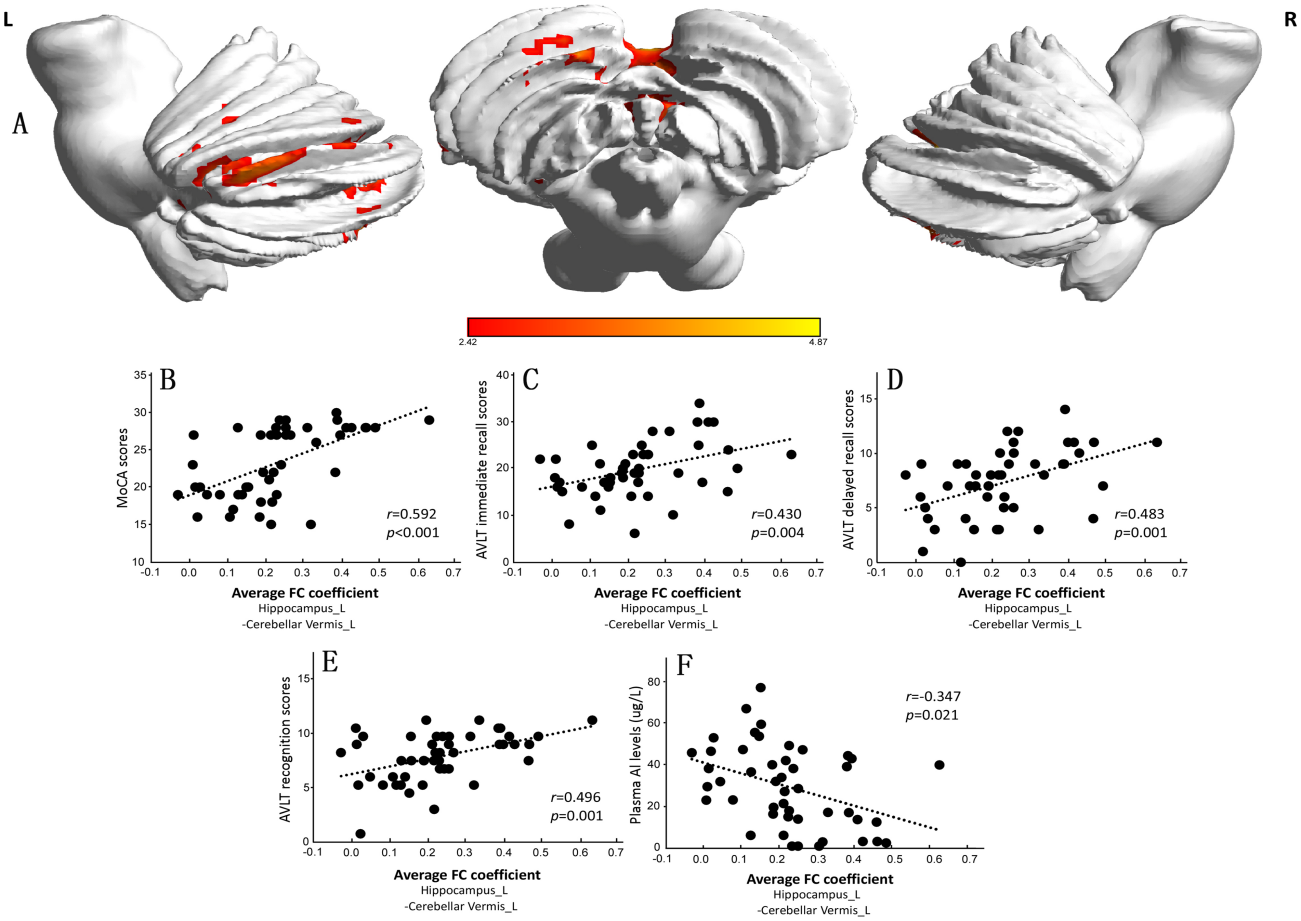
Significant differences in the functional connectivity (FC) of the left hippocampus between the normal control (NC) group and the cognitive impairment (CI) group. **(A)** Compared with those in the NC group, the FC in the left cerebellar vermis in the CI group was significantly lower. **(B–F)** The mean FC coefficient was calculated within the significantly altered brain area of each participant. The FC coefficient of the left cerebellar vermis was positively correlated with the cognitive scale score but negatively correlated with the plasma Al concentration. MoCA, Montreal Cognitive Assessment, Beijing version; AVLT, Auditory Verbal Learning Test.

We chose the left caudate nucleus and bilateral hippocampus as ROIs for the FC analysis of the whole brain. The results are presented in Supplementary Table S2. There was no significant difference in the FC of the left caudate between the CI and NC groups. The FC between the left hippocampus and the left cerebellar vermis in the CI group was lower than that in the NC group (Supplementary Table S2 and Figure 3A). The mean FC coefficient was subsequently calculated within the brain areas that were significantly altered in each participant, and the correlations of the FC coefficient with the MoCA score and plasma Al concentration were determined. The results showed that the FC coefficient between the left hippocampus and the left cerebellar vermis was positively correlated with the MoCA score (*r* = 0.592, *p* < 0.001; Figure 3B), AVLT immediate recall score (*r* = 0.430, *p* = 0.004; Figure 3C), AVLT delayed recall score (*r* = 0.483, *p* = 0.001; Figure 3D), and AVLT recognition score (*r* = 0.496, *p* = 0.001; Figure 3E) in the two groups but was negatively correlated with plasma Al concentration (*r* = −0.347, *p* = 0.021; Figure 3F).

**Figure 4:**
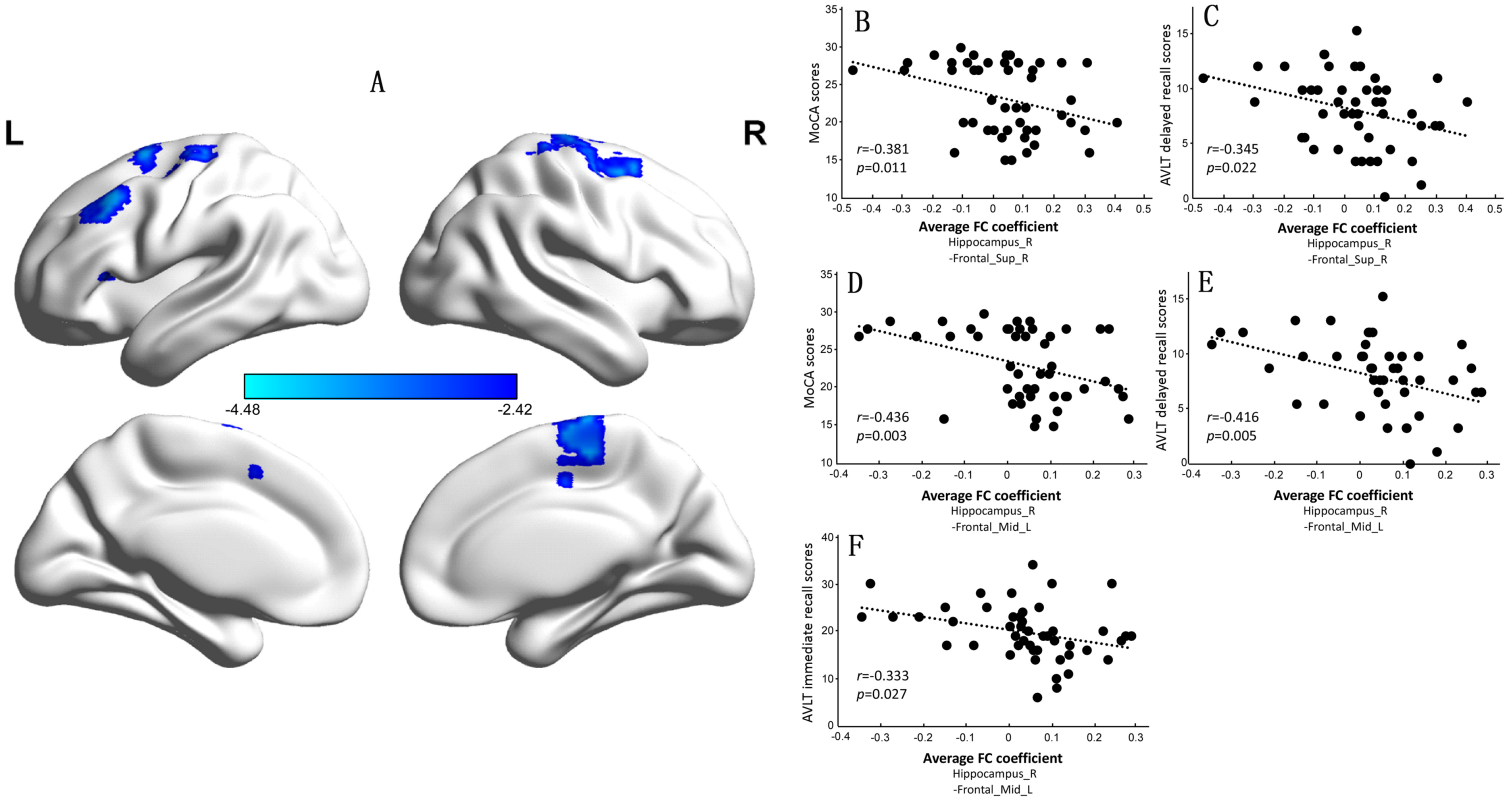
Significant differences in the functional connectivity (FC) of the right hippocampus between the normal control (NC) group and the cognitive impairment (CI) group. **(A)** Compared with those in the NC group, the FC in the left middle frontal gyrus and right superior frontal gyrus to the supplementary motor area was significantly greater in the CI group. **(B–E)** The mean FC coefficient was calculated within the significantly altered brain area of each participant. The FC coefficients of the left middle frontal gyrus and right superior frontal gyrus to the supplementary motor area were negatively correlated with the MoCA scores and AVLT delayed recall scores. **(F)** The FC coefficient of the left middle frontal gyrus was also negatively correlated with the AVLT immediate recall score. MoCA, Montreal Cognitive Assessment, Beijing version; AVLT, Auditory Verbal Learning Test.

However, compared with observations in the NC group, the CI group exhibited significantly increased FC between the right hippocampus and the left middle frontal gyrus (BA9) and between the right superior frontal gyrus and supplementary motor area (BA6) (Supplementary Table S2 and Figure 4A). The FC coefficients of the right superior frontal gyrus to the supplementary motor area and the left middle frontal gyrus were negatively correlated with MoCA scores (*r* = −0.381, *p* = 0.011; Figure 4B; *r* = −0.436, *p* = 0.003; Figure 4D) and AVLT delayed recall scores in the two groups (*r* = −0.345, *p* = 0.022; Figure 4C; *r* = −0.416, *p* = 0.005; Figure 4E). The FC coefficient of the left middle frontal gyrus was also negatively correlated with AVLT immediate recall scores in the NC and CI groups (*r* = −0.333, *p* = 0.027; Figure 4F).

### 3.5 GM and FC alterations mediated the Al-induced cognitive impairment

**Figure 5:**
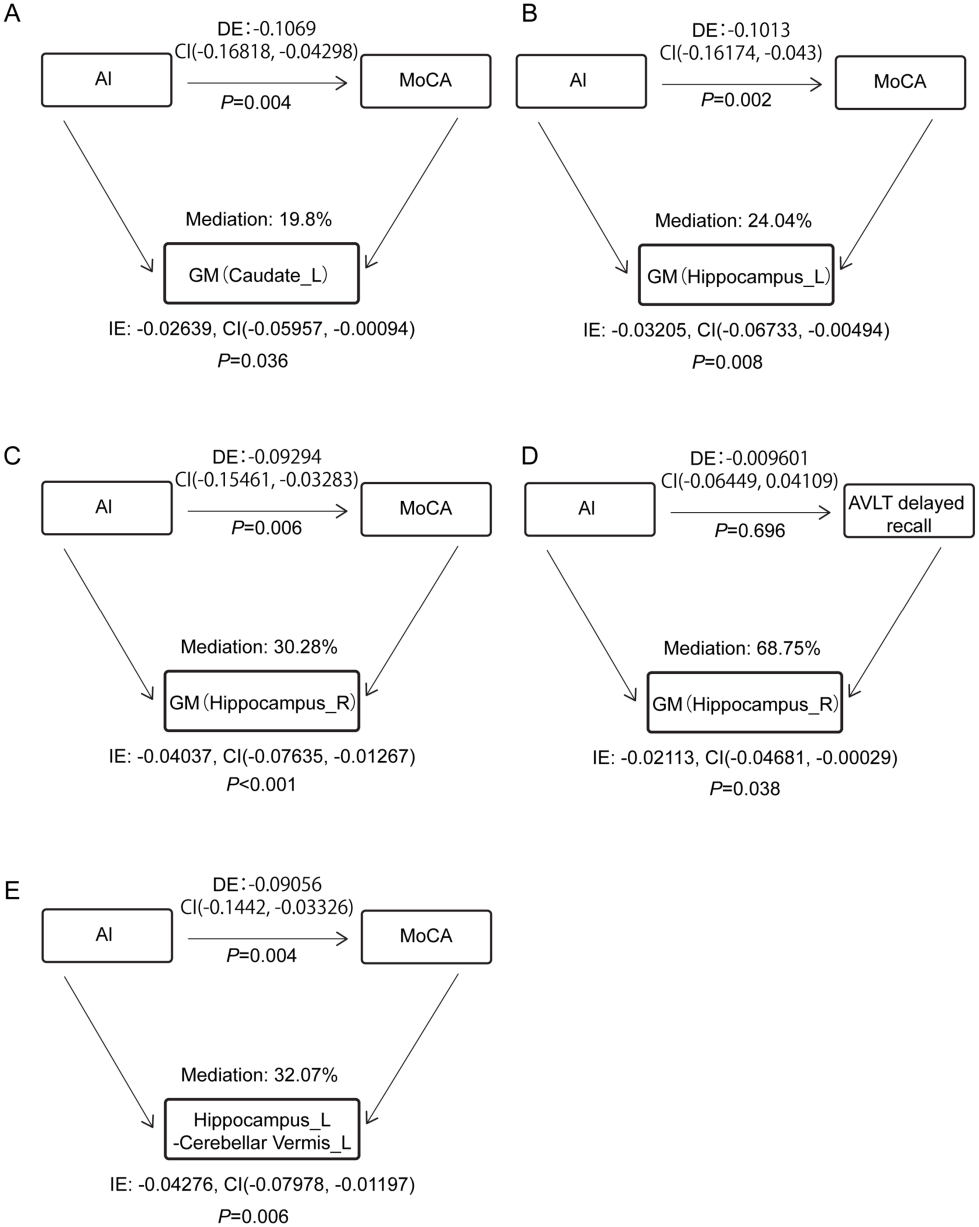
Estimated proportion of the association between Al and cognitive scales. **(A–C,E)** Al and MoCA. **(D)** Al and AVLT delayed recall. MoCA, Montreal Cognitive Assessment, Beijing version; AVLT, Auditory Verbal Learning Test; DE, the estimate of the direct effect; IE, the estimate of the indirect effect; Mediation, (IE/(DE + IE)); CI, confidence interval.

The association between Al and MoCA scores was mediated by the GM volume of the left caudate and bilateral hippocampus (*p* < 0.001), also mediated by the FC coefficient of the left hippocampus to the left cerebellar vermis (*p* < 0.001). In addition, the GM alteration of right hippocampus acted as indirect mediator of the association between Al and AVLT delayed recall scores (*p* = 0.038) (Supplementary Table S3 and Figure 5).

## 4 Discussion

In the present study, plasma Al concentration was negatively correlated with the MoCA score. We identified the brain regions with reduced GM volume in the CI group. GM volumes in these brain regions were positively correlated with cognitive scale scores. The FC between the bilateral hippocampus and the whole brain was altered and correlated with several cognitive scales. Moreover, plasma Al concentrations negatively correlated with bilateral hippocampal GM volume and FC coefficients between the left hippocampus and left cerebellar vermis. These brain regions can partly explain the effect of Al on cognitive impairment.

MRI studies of workers exposed to Al are lacking, and there has been little research on the structure and function of their brains. Our structural MRI results showed that areas with reduced GM volume were located in the bilateral hippocampus (CA1) and left caudate in the CI group. The hippocampus is located in the temporal lobe and is an important brain area that is mainly related to learning and memory. The CA1 region is the most sensitive to hypoxia and other types of damage (20–23). One study used VBM and a support vector machine model to analyze 77 sets of three-dimensional MRI data from patients with amnestic MCI; the results showed a significant reduction in GM volume in the left hippocampus (23). However, another study showed that the right hippocampal tail appeared to be important in patients with amnestic MCI (24). In this study, we observed a negative correlation between plasma Al concentration and bilateral hippocampal GM volume, indicating the importance of the hippocampus in pathological processes. This is consistent with a reduction in caudate nucleus volume during the progression of MCI to AD (25, 26).

Our correlation analysis showed that the volumes of the bilateral hippocampus and left caudate nucleus were positively correlated with the MoCA score. Furthermore, GM volumes in the left caudate and right hippocampus were positively correlated with immediate and delayed recall scores on the AVLT, respectively. The memory performance of patients with MCI is related to the volume of the hippocampal area, and delayed memory impairment is related to a reduction in the volume of the hippocampal CA1, CA4, and dentate gyrus (27). In our previous animal study (28), the axons of hippocampal CA1 neurons were beaded, and the number of dendritic spines decreased in Al(mal)_3_-exposed rats; moreover, long-term potentiation was suppressed in the hippocampal CA1 region, which may be the pathophysiological basis of cognitive decline, especially memory impairment.

A possible pathway from which pollutants can exert their widespread detrimental effects on cognition might be represented by the “human brain connectome” (16). FC may reflect interrelations between different brain regions and tissues. Some fMRI studies (29, 30) have emphasized abnormal FC between the hippocampus and other brain regions in patients with AD or MCI. The hippocampus has extensive connections with the cerebral cortex and other subcortical structures. In the present study, we chose the different brain regions mentioned above as ROIs to study changes in FC in the whole brain. Compared with observations in healthy controls, the FC between the left hippocampus and left cerebellar vermis decreased. Recently, increasing attention has been paid to the role of the cerebellum in non-motor functions (31, 32). The cerebellum is involved in language, executive function, spatial processing, and other cognitive processes (33, 34). Studies revealed that the anterior cerebellar lobe participates in perceptual motor function and is activated during the process of maintaining verbal working memory (35–38). In our study, the FC between the hippocampus and cerebellum decreased, and the FC coefficient of the left cerebellar vermis was positively correlated with the MoCA, AVLT immediate recall, AVLT delayed recall, and AVLT recognition scores, demonstrating that the lower the cognitive score, the weaker the FC between the two brain regions. Furthermore, plasma Al concentrations were negatively associated with the FC coefficient from the left hippocampus to the left cerebellar vermis. The decrease in FC between the left hippocampus and left cerebellar vermis may be a neuroimaging mechanism of cognitive decline in Al workers.

However, compared with that in the NC group, the CI group exhibited significantly increased FC between the right hippocampus and left middle frontal gyrus (BA9)/the right superior frontal gyrus and supplementary motor area (BA6). These areas are located in the frontal lobe and are mainly responsible for high-level psychological activities and cognitive functions, such as emotion, decision-making, reasoning, prediction, and short-term memory. FC between the hippocampus and the frontoparietal network increases in patients with AD (39, 40). The FC coefficients of the left middle frontal gyrus and right superior frontal gyrus with the supplementary motor area were negatively correlated with the MoCA and AVLT delayed recall scores, whereas the FC coefficient of the left middle frontal gyrus was negatively correlated with the AVLT immediate recall scores. Therefore, changes in FC may be clinically significant. To maintain cognitive efficiency, enhanced FC may compensate for decreased function in other brain regions. These results indicate that brain FC was disrupted, and brain function was imbalanced in the CI group. Based on our previous study (41), we observed that synaptic plasticity in the hippocampus of rats exposed to aluminum changed, and these findings complemented the population outcomes. Aluminum may affect the structure and FC of the hippocampus with other brain regions by enabling hippocampal neuronal death and altering hippocampal synaptic plasticity. The results of the mediation effect analysis also support this point, can partly explain the effect of Al induced CI.

In our study, cranial magnetic resonance scans were performed on Al workers, and structural and functional brain changes were observed. This study had several limitations. First, our sample size was small, and additional participants must be recruited to obtain more precise data. Second, this was not a longitudinal study, and dynamic observations are necessary. Third, multimodal MRI should be used for further research on neuroimaging mechanisms, such as brain network, integrity of the blood brain barrier, even activation of microglia, and multiple-angle analysis methods should be used. Finally, an additional group of participants matched to the HC participants who did not experience occupational aluminum exposure would add important and essential evidence to the conclusion.

## 5 Conclusion

Our study found that the CI group exhibited GM impairment and altered hippocampal FC with the left cerebellar vermis, left middle frontal gyrus, and right superior frontal gyrus relative to the supplementary motor area. These disruptions seem to be related to a decrease in hippocampal FC or a compensatory enhancement. Changes in left hippocampal structure and function correlated with increases in plasma Al concentration and decreases in the MoCA score. The present findings provide evidence of the pathophysiological process of CI in Al workers and may lead to the use of imaging biomarkers and provide a theoretical basis for mechanistic research.

## Data Availability

The original contributions presented in the study are included in the article/Supplementary material, further inquiries can be directed to the corresponding author.
