# Supplementary material for: Alterations of gray matter volume and functional connectivity in patients with cognitive impairment induced by occupational aluminum exposure: a case-control study
Source: Front Neurol. 2025 Jan 7;15:1500924. doi: 10.3389/fneur.2024.1500924 (PMC11747109; doi:10.3389/fneur.2024.1500924)
Supplement: Supplementary file 1 [file Data_Sheet_1.docx]

**Supplemental table 1** Regions of reduced GM volume in CI

| **Brain region** | **Cluster size** | **Peak T value** | **MNI coordinates** | | |
| --- | --- | --- | --- | --- | --- |
|  |  |  | **X** | **Y** | **Z** |
| Left caudate | 729 | 3.988 | -12 | -5 | 26 |
| Right hippocampus | 644 | 4.520 | 18 | -38 | 9 |
| Left hippocampus | 448 | 3.970 | -20 | -41 | 6 |

*P* < 0.001 at voxel level and alteration significant at the cluster level (*P* < 0.05, FWE corrected) will be reported.

**Supplemental table 2** Significant inter-group differences identified in the VBM-seed functional analysis

| **Brain region** | **Cluster size** | **Peak T value** | **MNI coordinates** | | | |
| --- | --- | --- | --- | --- | --- | --- |
|  |  |  | **X** | | **Y** | **Z** |
| **CI < NC (ROI: left hippocampus)** | | | | | | |
| Left cerebellar vermis | 534 | 4.873 | 0 | -72 | | -27 |
| **CI > NC (ROI: right hippocampus)** | | | | | | |
| Left middle frontal gyrus (BA9) | 393 | -4.476 | -45 | 18 | | 51 |
| Right superior frontal gyrus and  supplementary motor area (BA6) | 315 | -3.886 | 15 | -9 | | 51 |

*P* < 0.01 at voxel level and alteration significant at the cluster level (*P* < 0.05, FWE corrected) will be reported.

**Supplemental table 3** Mediating analysis of brain changes between AI exposure and cognitive scales

| **M** | **Y** | **Exposure Vs. mediator** | **Mediator Vs. outcome** | **indirect effect** | **direct effect** | **proportion mediated(%)** |
| --- | --- | --- | --- | --- | --- | --- |
| GM（Caudate_L） | MoCA | -0.0008824 (-0.00151,-0.00025) | -0.1069 (-0.17403,-0.03982) | -0.02639 (-0.05957,-0.00094) | -0.1069 (-0.16818, -0.04298) | 19.8 |
| GM（Hippocampus_L） | MoCA | -0.0006032 (-0.00105,-0.00016) | -0.1013 (-0.16624,-0.03629) | -0.03205 (-0.06733,-0.00494) | -0.1013 (-0.16174, -0.043) | 24.04 |
| GM（Hippocampus_R) | MoCA | -0.0004864 (-0.00085,-0.00013) | -0.09294 (-0.15459,-0.03129) | -0.04037 (-0.07635, -0.01267) | -0.09294 (-0.15461, -0.03283) | 30.28 |
| GM（Hippocampus_R） | AVLT delayed recall | -0.0004864 (-0.00085,-0.00013) | -0.009601 (-0.05997,0.04076) | -0.02113 (-0.04681, -0.00029) | -0.009601 (-0.06449, 0.04109) | 68.75 |
| Hippocampus_L to cerebellar vermis_L | MoCA | -0.002695 (-0.00479, -0.0006) | -0.09056 (-0.14973,-0.03139) | -0.04276 (-0.07978, -0.01197) | -0.09056 (-0.1442, -0.03326) | 32.07 |

*P* < 0.01 ,Covariates include age and education
